# Wound Antiseptics and European Guidelines for Antiseptic Application in Wound Treatment

**DOI:** 10.3390/ph14121253

**Published:** 2021-12-02

**Authors:** Zuzanna Łucja Babalska, Marzena Korbecka-Paczkowska, Tomasz M. Karpiński

**Affiliations:** 1 Chair and Department of Medical Microbiology, Poznań University of Medical Sciences, Wieniawskiego 3, 61-712 Poznań, Poland; z.babalska@gmail.com; 2 Medi-Pharm, os. Konstytucji 3 Maja 14/2, 63-200 Jarocin, Poland; mkorbecka@wp.pl

**Keywords:** antiseptics, antisepsis, wounds, treatment, octenidine, polihexanide, povidone-iodine, nanosilver, sodium hypochlorite

## Abstract

Issues arising in wound healing are very common, and chronic wound infections affect approximately 1.5% of the population. The main substances used in wound washing, cleansing and treatment are antiseptics. Today, there are many compounds with a known antiseptic activity. Older antiseptics (e.g., boric acid, ethacridine lactate, potassium permanganate, hydrogen peroxide, iodoform, iodine and dyes) are not recommended for wound treatment due to a number of disadvantages. According to the newest guidelines of the Polish Society for Wound Treatment and the German Consensus on Wound Antisepsis, only the following antiseptics should be taken into account for wound treatment: octenidine (OCT), polihexanide (PHMB), povidone-iodine (PVP-I), sodium hypochlorite (NaOCl) and nanosilver. This article provides an overview of the five antiseptics mentioned above, their chemical properties, wound applications, side effects and safety.

## 1. Introduction

The treatment of wounds, especially chronic wounds, is a major challenge for medical personnel. It is estimated that chronic wounds are a problem in as much as 1.5% of the population [1,2]. In Poland, this problem affects almost 500,000 patients, while approximately 4.5 million people are affected in the USA. In wound care, comprehensive local treatment is extremely important, including the use of antiseptic preparations, which constitute a key element of the therapeutic process, as well as specialized dressings [3,4].

Looking back to history, at the beginning of the 19th century, hospitals did not have sanitary equipment and most lacked even separate operating theatres. Doctors did not use gloves and washed their hands after surgery rather than before. Post-operative mortality at that time reached 75% [5]. Ignaz Semmelweis observed that the maternity ward in which doctors and students worked had a mortality rate of 20%, while the maternity ward in which midwives worked had a mortality rate of only 3%. Students came to the ward immediately after the autopsy without washing their hands to examine the women. Semmelweis ordered all doctors and students leaving the morgue to wash their hands in chlorinated water; shortly thereafter, post-partum mortality fell [6]. Joseph Lister, after reading an article by Louis Pasteur regarding the fermentation of milk under the influence of microorganisms, concluded that a similar phenomenon occurs in infected wounds. He decided to use carbolic acid as an antiseptic [7]. It quickly became apparent that carbolic acid is very irritating to the respiratory tract and that cutaneous exposure causes skin irritation. Soon thereafter, other antiseptics were invented, with notable examples including iodoform and potassium permanganate [1].

## 2. General Definitions

The term antiseptic is derived from the Greek words: anti–anti and sepsis–rot; therefore, antiseptic literally means anti-rot. Broadly, antiseptics are chemicals applied to the skin or other living tissues with the aim of inhibiting microbial growth. This action does not involve the destruction of spores [8]. A concept related to antisepsis is the notion of asepsis. These terms should be distinguished. Asepsis aims to ensure the sterility of rooms, tools, medicines, dressings, and other objects, thus preventing microbial contact with wounds in the first place. Asepsis includes physical and chemical processes of microbial destruction.

Disinfection is a process that eliminates microorganisms, with the exception of bacterial spores, on inanimate objects. Disinfection is divided into three levels:

− low-level disinfection, which consists of reducing the vegetative forms of bacteria (except *Mycobacterium tuberculosis*), enveloped viruses and fungi. This category of disinfectant includes 3% hydrogen peroxide, quaternary ammonium compounds, diluted phenolics and glutaraldehyde.
− Intermediate-level disinfection is the process of reducing all species of bacteria in the vegetative form (including Tbc), viruses (enveloped and non-enveloped) and fungi. Disinfectants belonging to this category include phenolics, iodophor, alcohols, and chlorinated compounds.
− High-level disinfection—in addition to the above, disinfectants in this class reduce spores, i.e., spore forms. This category of disinfectants includes ≥2% glutaraldehyde, 7.5% hydrogen peroxide, hypochlorite, and hypochlorous acid [8,9].

Sterilization is a technological procedure that removes microorganisms and their spores from objects (e.g., medical instruments, medicines). It can be done by physical methods (e.g., X-rays, cooking, annealing, UV irradiation, tanning, dry hot air, exposing steam to pressure, flame) as well as chemical methods [9].

## 3. Antiseptics

### 3.1. The Difference between an Antiseptic and a Disinfectant

In pharmacology, a distinction is made between antiseptics and disinfectants. Disinfectants are chemicals that destroy microorganisms in vegetative forms. Some compounds belonging to the high-level disinfectants act additionally against spore forms. They also prevent microbial reproduction. By means of disinfection (i.e., through the use of agents classified as disinfectants), it is possible to obtain aseptic conditions. Disinfectants are used to disinfect rooms, objects (e.g., sanitary facilities, walls, furniture, floors, instruments), or components of the natural environment (e.g., watercourses, soil) [9]. Antiseptics are substances that kill microbes on the surface of the skin and mucous membranes. They should be characterized by anti-bacterial, anti-fungal, and anti-viral effectiveness as well as safety in use [8].

### 3.2. The Use of Antiseptics

Antiseptics are used on the surfaces of the body, mainly on the skin, mucous membranes and surface wounds. The preventative and medicinal uses of antiseptics can be distinguished [10]. These agents are often used in hospitals and healthcare facilities to control the risk of infection as well as to prevent nosocomial infections [11]. Antiseptics are applied by medical staff to decontaminate the skin of the hands, pre-operatively clean the skin of the surgical site, and cleanse chronic and acute wounds. They are also used to treat open wounds and sometimes infections of the skin [3,4]. The general public increasingly uses them for fear of microbial contamination of food and industrial goods. Nowadays, with the general heightened sense of awareness regarding the dangers of certain microbes, such as SARS-CoV-2, they are used in homes and workplaces to reduce the spread of infections [11].

### 3.3. Features of Antiseptics

An antiseptic should have broad antimicrobial activity (viruses, fungi, bacteria), which also covers antibiotic-resistant microorganisms such as methicillin-resistant *Staphylococcus aureus* (MRSA) or vancomycin-resistant enterococci (VRE). Many of these substances can be used alone or in products containing several compounds with antimicrobial activity that differ in their efficacy and spectrum against microorganisms [11]. It should be noted that in prophylactic antiseptics, the substances are applied once or a few times over a short period of time. This form of antisepsis is strong and fast-acting. However, with therapeutic antisepsis, the agents are used continuously and often for longer periods of time. In addition, their low cytotoxicity and good antimicrobial activity are important [12]. Antiseptics should be generally safe to use, not cause allergic reactions or pain, and should not be toxic, carcinogenic or mutagenic. Additionally, they should achieve effective antimicrobial concentrations at the site of action while not leading to the development of resistance. Depending on the application (destination), they should have appropriate chemical and physical properties such as color, smell, consistency and taste. They also cannot interfere with wound healing. Additionally, they should show activity against bacterial biofilm [10,13]. The Biocompatibility Index (BI) helps choose the right antiseptic. A value of BI > 1 indicates that the product is characterized by broad-spectrum activity against microorganisms and a low level of cytotoxicity against fibroblasts or keratinocytes; therefore, its use does not adversely affect the healing process. The substances meeting these requirements are octenidine (BI = 1.7–2.1) and polyhexanide (BI = 1.4–1.5) [12,13].

In addition, research by Pitten et al. revealed that the bactericidal properties of antiseptics change under the influence of substances such as exudate in the wound, albumin, mucins, or blood. Therefore, using a specific antiseptic depends on the target of its action and the presence of interfering substances [14].

### 3.4. Old Antiseptics

From the 19th century, many compounds were used in wound antisepsis. First was carbolic acid (phenol), isolated by Runge in 1834 and used as an antiseptic by Joseph Lister. He applied bandages soaked in aqueous phenol solution to the wounds of patients. In addition, he ordered this agent to be used for disinfecting hands and the operating field [7]. We also include other compounds in the old antiseptics, like boric acid, ethacridine lactate, potassium permanganate, hydrogen peroxide, iodoform, or ethanolic iodine solution. Each one of them has numerous disadvantages, for example, poor antimicrobial efficacy, growing resistance among bacteria, poor ability to penetrate biofilms, tissue intolerance, eliciting pain, inducing allergic responses, cytotoxicity and/or carcinogenicity, and inactivation under the influence of protein loads [4,15,16]. For example, iodoform was used, among others, in dentistry, but it is strong irritant, induces allergic reactions and is cytotoxic to macrophages and epithelial cells [17,18]. On the other hand, iodine water-alcohol tincture has been widely used in the past. A 5% solution was used in humans as an anti-bacterial and anti-fungal agent, while a 10% solution was used in veterinary medicine. Unfortunately, it tends to cause chemical burns, has a short period of activity and is relatively unstable [9,19]. For many years, antiseptics were used as dyes in solution, such as crystal violet, brilliant green, malachite green and methylene blue. However, many researches have indicated the mutagenic and carcinogenic activity of dyes [20,21,22,23,24]. In Italy it is produced antiseptic with 2% eosin; however, in PubMed, we did not find any information about the antimicrobial activity of eosin.

## 4. Antisepsis of Wounds

### 4.1. Octenidine Dihydrochloride

Octenidine dichloride (OCT) is a cationic, non-volatile, surface-active compound. It shows stability at pH 1.6–12.2. It does not change its properties under the influence of light and can be stored at room temperature. This compound can be sterilized at 130 °C with steam [12,25]. OCT has two cationically active centers. This feature allows OCT to bind to negatively charged surfaces easily. This includes the envelopes of microbial cells and membranes of eukaryotic cells. OCT has an affinity for polysaccharides and phospholipids. On account of this, OCT applied to the skin, mucous membranes or wounds provides activity against bacteria, fungi and enveloped viruses. OCT in concentrations 0.05–0.1% is microbicidal in just 1 min against tested bacteria and fungi, including *Staphylococcus aureus*, *Pseudomonas aeruginosa* and *Candida albicans* [13,16,26]. To date, no confirmed cases of acquired resistance to this antiseptic have been reported [27].

#### 4.1.1. Application

OCT is used in the prevention and treatment of local infection in the form of gel and liquid [28]. OCT is present in products used for antisepsis of wounds and mucous membranes, including the oral cavity, and with the addition of alcohol to disinfect the skin before diagnostic and surgical procedures. It is also found in products for handwashing among both patients and medical staff [27]. Products containing this antiseptic usually have an OCT concentration of 0.05–0.1% [3,29]. Due to the excellent antimicrobial activity of 0.1% OCT in combination with phenoxyethanol, it is suitable for treating acute, traumatic and infected wounds, including those colonized by multi-resistant strains. For rinsing, washing and cleaning of extensive wounds, 0.05% OCT with ethylhexylglycerin content is recommended [30]. Gel with OCT is suitable for burns and is better than PVP-I and silver in this scenario [28]. In addition, safety and efficacy in the use in newborns and premature babies, as well as pregnant and breast-feeding women, have been confirmed [27,31,32]. Moreover, research has shown that the use of OCT for coating tracheostomy tubes reduces infection in artificially ventilated patients. However, applications in ready-to-use products have not yet been found [33]. OCT is also used to coat surgical sutures [34]. The addition of a surfactant to products containing OCT has a strong and effective anti-biofilm effect [10]. There are no contraindications in using products containing OCT with specialist dressings containing silver compounds or polyhexanide [29].

#### 4.1.2. Safety

OCT does not penetrate mucous membranes, the skin, placental barrier or wounds [12,35]. No side effects have been observed when using octenidine on the skin [36]. The fact that it is not absorbed through the skin, as is the case with alcohol-based antiseptics, supports its use in newborns and premature babies [37]. For this reason, octenidine is approved for topical use and no toxic systemic or local effects are to be expected in treated patients [12,25]. In addition, OCT has lower cytotoxicity to fibroblasts and human epithelial cells rather than chlorhexidine used in mouthwash [38]. Studies by Muller and Kramer [12] have shown that OCT is more toxic to microorganisms than to mouse fibroblasts, thereby suggesting that the benefit provided by its antimicrobial properties outweighs its potential cytotoxic effects.

OCT is intended for external use only, as it cannot be used for injections, for rinsing the peritoneal cavity or in the administration to CNS structures. In order to ensure safety when using OCT, drainage should be provided [28,30]. A clinical trial confirms the possibility of using OCT for up to 3 months in difficult-to-heal wounds [36].

### 4.2. Povidone-Iodine (Polyvinylpyrrolidone-Iodine; PVP-Iodine)

Today, the most commonly used iodine compound is polyvinylpyrrolidone-iodine (PVP-I), which is used in wound products and ointments. PVP-I was introduced as an antiseptic by Shelanski and Shelanski in 1956 [39]. It is formed by joining iodine molecules with polyvinylpyrrolidone and is water-soluble [5]. In PVP-I, povidone acts as an iodine carrier and is capable of absorbing iodine and transporting it, but does not react with the iodine. However, the active substance in this compound is iodine itself. 5–12% PVP-I solutions are used for antiseptic purposes [35]. It should be noted that the concentration of free iodine increases as the preparations are diluted. Through dilution, iodine and carrier (PVP) bonds are weakened, contributing to an increase of free iodine in the solution. Dilute solutions (0.1–1%) work faster than the concentrated 10% solution [40]. PVP-I shows bactericidal, fungicidal and virucidal activity against enveloped viruses within 1–5 min [40,41]. The 1% solutions of PVP-I also has a sporicidal activity against spores of *Bacillus subtilis*, but the required action time for 99% kill is from 28 to 93 min [42].

#### 4.2.1. Application

At present, PVP-I is mainly used in the form of liquids and ointments, ointments at concentrations of 7.5% and 10%, respectively [30]. However, it also comes in the form of a powder. The PVP-I solution is used as a medicinal product for antiseptic treatment of small superficial wounds of the skin and mucous membranes [43]. It is the agent of choice for gunshot wounds, stab wounds and bites. In the case of chronic wounds, it is not recommended due to its cytotoxicity and the lack of synergistic action with silver dressings. It can also be used in post-operative wounds and diabetes-associated foot damage [44]. PVP-I has also occasionally been used for the non-surgical treatment of periodontitis; however, this treatment is rarely used die to its brown color. Alcohol solutions of PVP-I are used to disinfect the skin before surgery. Due to possible skin burns and its alcohol content, this antiseptic is contraindicated in children under 7 years of age. However, solutions of PVP-I are suggested for use in newborns as a bactericide before invasive procedures [45]. In addition, the use of intraoperative rinse with iodine reduces the incidence of infections, especially in spine surgery (3.5% betadine) [46], breast surgery (4% PVP-I solutions) [47], abdominal surgery (Betadine gel) [48], and arthroplasty (3.5% Betadine) [49]. A recent study revealed that the short antimicrobial action of PVP-I is ineffective against microbial biofilms [50]. PVP-I preparations are recommended for sharp, cut and lacerated wounds; however, they are not recommended for chronic wounds and those presenting with a difficulty in healing due to the low BI index (<1). PVP-I preparations should not be combined with specialist dressings containing silver compounds, which additionally limits their use in the treatment of chronic wounds [28].

#### 4.2.2. Safety

PVP-I is less irritating to the skin in comparison to ordinary iodine solutions. In addition, PVP-I is less toxic and therefore, accidental ingestion is less dangerous than ordinary iodine solutions. No mutagenic, neurotoxic, teratogenic or carcinogenic effects have been observed to this day [28,51]. Research by Zhou et al. [52] showed no cytotoxic activity on fibroblast cells, at a concentration of 0.45% [53]. PVP-I should not be used in newborns, young children, pregnant women or during lactation. Thyroid diseases and iodine radiotherapy are also contraindications. Even if no thyroid disease has been reported, povidone-iodine should not be used for more than 7 days, as thyroid disorders may occur [28]. No tissue damage was observed in several studies examining the treatment of chronic wounds with PVP-I [52,54].

### 4.3. Polihexanide

Polyhexamethylene hydrochloride biguanide (polyhexanide, PHMB) was first synthesized in the 1950s in the laboratories of ICI Ltd. [55] It is a cationic biguanide polymer. PHMB binds to negatively charged phosphate phospholipid groups that are a component of the bacterial cell wall. By sinking non-polar segments of the molecule into the hydrophobic interior of the cell membrane, it results in membrane dysfunction. PHMB increases the distance between the lipid molecules of the membrane and affects the proper functioning of ion pumps, various enzymes and bacterial cell receptors. Furthermore, it stiffens the liquid bilayer membrane, leading to an increase in its permeability. The accumulation of adverse effects induced by PHMB ultimately leads to the disruption of the cell wall and membrane and the death of microorganisms exposed to the antiseptic [35,55]. To date, no confirmed cases of acquired resistance to this antiseptic have been reported. It is colorless, odorless and non-corrosive, and is soluble in water and alcohol [35]. PHMB is bactericidal and fungicidal within 15–30 min [4,28].

#### 4.3.1. Application

PHMB has been used in the non-medical consumer industry for about 40 years [55] in a wide range of antimicrobial applications such as anti-bacterial treatment of textiles, the preservation of cosmetics, industrial disinfection and the sanitation of recreational water [56]. For medical applications, it was introduced by the Swiss surgeon Willenegger. In the 1990s, he used it to treat wounds locally [57]. Polihexanide is available in the form of a liquid or gel in commercial preparations in combination with Ringer’s solution, betaine or poloxamer. This agent is present in concentrations of 0.02%, 0.04% and 0.1%. PHMB impregnated dressings are also available [4,28,58]. As reported by Kramer et al. [28], such dressings completely eliminate *Staphylococcus epidermidis* strains within 24 h. PHMB is active against MRSA and VRE strains. It is better at healing wounds than silver and PVP-I, because it does not inhibit the reepithelialization process and inhibits proteolytic enzymes. For this reason, it is recommended in the treatment of epithelial lesions and second-degree burns [30,59,60]. Recently, a study has emerged pointing to the supporting role of PHMB in the treatment of bacterial vaginosis [61].

#### 4.3.2. Safety

Despite the high affinity of PHMB with microbial cells, this substance has limited effects on human and animal cells. Polihexinide has a large margin of safety in clinical use [55]. Studies on the human skin have shown low absorption of this agent via the epidermis [62]. In addition, studies have shown a low probability of occurrence of allergic reactions. Allergy to PHMB is associated with dermatitis, old age and occupational exposure [63]. PHMB is irritating to the respiratory tract at a concentration of 264 mg/m^3^ [62]. PHMB has a high affinity for tissue structures. Therefore, long-term use should be avoided. At present, evidence for this untoward aspect of PHMB use is supported by the appearance of grayish inert tissue after using PHMB alone or in combination with betaine [28]. Animal studies have shown an increase in the incidence of vascular tumors, mainly liver hemangiosarcomas, in doses started from 28 mg/kg. According to the Committee for Risk Assessment (RAC), PHMB is considered potentially carcinogenic [62].

### 4.4. Sodium Hypochlorite (NaOCl)

Active chlorine is the active substance released from aqueous sodium hypochlorite solutions. In water, NaOCl hydrolyses to hypochlorous acid, which is then kept in equilibrium with chlorine. This means that the ratio of chlorine (Cl_2_), hypochlorous acid (HClO) and ClO- hypochlorite ion depends on the temperature and pH of the aqueous environment. Sodium hypochlorite is an unstable substance as a pure salt. For this reason, it is produced and used as an aqueous solution with a pH above 11 at 20 °C [64,65]. By nature, sodium hypochlorite solutions are considered unstable. Their stability depends on several factors, including the concentration, storage temperature, and availability of light and air. The rate of decomposition of NaOCl increases as the pH decreases. Most of the published studies are conducted in vitro, and it should be remembered that the effectiveness of sodium hypochlorite (NaOCl) also depends on conditions such as protein load, and wound exudate [28,35,65].

#### 4.4.1. Application

It is widely used by private consumers as well as by large industrial operations. It is commonly used in the cleaning, washing and dyeing fabrics, as well as in perfumes, cosmetics, body care products, and the treatment of drinking water and wastewater. NaOCl is also widely used in surface disinfection. In agriculture, it is used as a seed and soil treatment agent [35].

In studies originating in Japan, sodium hypochlorite was used as a hand exfoliant (0.01–0.05%), an antiseptic for skin and mucosal wounds (0.005–0.01%), a tool disinfectant (0.0125–0.05%), a surgical site antiseptic (0.01–0.05%), and a surface disinfectant (0.0125–0.05%) [66]. Solutions containing hypochlorous acid are used in wound healing, to wash traumatic, acute and chronic wounds without drainage, e.g., the peritoneal cavity. Kramer et al. [28] demonstrated the legitimacy of using these compounds in acute and chronic wounds. Products are available in the form of gels or liquids. Hypochlorites used in wound healing have low concentrations of 0.004% to 0.03% [30]. These agents are well tolerated at the aforementioned low concentrations. Hypochlorite concentrations above 0.05% have a significant effect on the inhibition of fibroblasts, an undesirable effect in wound healing [28]. Due to its ability to dissolve tissues, sodium hypochlorite is used in dentistry as an endodontic irrigator, in concentrations 0.5–5.25% [67].

#### 4.4.2. Safety

Absorption through the skin is negligible if the cell layer is intact. No systemic effects have been demonstrated with the topical use of NaOCl. The same applies to the inhalation of sodium hypochlorite. However, sodium hypochlorite at a concentration higher than 10% is corrosive to the skin and eyes. In contrast, irritation of the skin and necrosis is observed in concentrations 5–10%. Allergic reactions and dermatitis may be observed in patients using NaOCl [68,69,70]. Persons with hypersensitivity to sodium hypochlorite may experience symptoms such as swelling, pain, breathing problems [71,72,73]. Many studies have shown that sodium hypochlorite can lead to tissue necrosis in a concentration-dependent manner [19,74].

### 4.5. Nanosilver

Silver compounds have been used in wound care since the 17th century [75]. Silver compounds result in the modification of bacterial membrane proteins, resulting in the leakage of H+ ions and thus a reduction in the proton-motive force. Furthermore, silver compounds impair the permeability of the cell membrane, leading to the death of the microorganism. The effect of silver on any membrane protein explains its broad antimicrobial spectra [76].

Currently, mainly are used silver nanoparticles, which are produced using two methods: chemical silver reduction and laser ablation. However, in the first method, it is not easy to regulate the size of the nanoparticles formed, as is the case with the latter method. The biochemical, physical and antimicrobial properties depend on the size of the silver nanoparticles. In addition, nanoparticles suspended in solution have different magnetic, optical and catalytic properties [77]. The size of the nanoparticles affects their antimicrobial activity. Nanoparticles having greater surface contact with microorganisms and allow for increased interactions between both [78,79]. The electronic effects produced by nanoparticles smaller than 10 nm increase their reactivity and affect interactions with bacteria [78]. Research by Pal et al. [79] shows that the shape of the particles also affects antimicrobial activity. Triangular particles have better antimicrobial performance than spherical or rod-shaped particles.

Silver nanoparticles interact with the bacterial cell membrane and penetrate the interior of the cell. They react with sulfur and phosphorus, which are found in the membrane and DNA of bacteria. These particles also induce the dysregulation of various elements of the respiratory chain and inactivate enzymes. As a result of all these interactions, they lead to the death of bacterial cells [78,80,81].

#### 4.5.1. Application

Many medical devices, with the exception of dressings, coated with metallic silver or silver ions have proved to be insufficient in their antimicrobial activity for clinical use [82]. Due to signs of the emergence of resistance to silver by some bacteria, there has been increased interest in silver nanoforms with improved antimicrobial activity [83,84]. At present, nanoparticles are candidates for coating medical devices. Furno et al. [85] showed that coating devices with nanoparticles is effective and prevents infection. However, the antimicrobial activity decreases as the devices are washed. In addition, silver nanoparticles are used to coat surgical masks [86]. Gels and dressings containing silver are used to treat chronic wounds and prevent their infection [87]. Animal studies have shown better wound healing, reduced scarring, and a better appearance of a healed wound [88]. In addition, silver nanoparticles can be used for water treatment and are effectively removed with a magnetic field, thus eliminating environmental pollution [89]. The Polish Wound Treatment Society allows the use of silver-based preparations for the treatment of wounds at risk of infection and critically colonized wounds as a second-line measure. Society states that it should be used only up to the point at which the infection is cleared and not thereafter [30].

#### 4.5.2. Safety

Regardless of the form in which we use silver ions (whether they are nanoparticles, inorganic or organic compounds), the insoluble silver sulfides can always precipitate in the dermis or wound. This manifests in the form of argyria, i.e., black and blue discoloration [90,91].

Research by Liu et al. has shown that the toxicity of silver nanoparticles depends on their size. Smaller nanoparticles penetrated cells more easily and caused greater changes in their morphology. Silver nanoparticles cause damage to the cell membrane, induce cell cycle arrest, and the accumulation of reactive oxygen species and apoptosis [92]. Animal studies have confirmed the toxic effects of silver nanoparticles on organs such as the liver, kidneys, spleen and lymph nodes. Silver accumulates in individual organs and leads to their destruction [93,94]. At times, even changes in animal behavior were observed [95]. Research by de Lima et al. [96] and Munger et al. [97] showed that human cells are less susceptible to the toxic effects of silver nanoparticles. This suggests that the effects seen in animal studies may not occur in humans, and future studies should be conducted to confirm this finding. Oral exposure is more dangerous than external exposure.

After ingestion, a decrease in body mass, biochemical changes in the blood and altered liver parameters are observed. The most vulnerable organ is the liver due to its high detoxification potential. However, the penetration of silver nanoparticles from the lumen of the intestine into the bloodstream can negatively affect all organs. Nano-silver is excreted in bile and urine [98]. However, studies have shown that topical application to the skin is less toxic. External administration does not cause accumulation in key human organs and is mainly excreted in the feces. Topical preparations are therefore safe to use. However, in the case of medical use of silver nanoparticles, consideration should be given to the dose, particle size, route of administration, and exposure time [99].

Anti-bacterial activity (MICs) of the above-described antiseptics against *S. aureus* and *P. aeruginosa* is presented in Table 1.

## 5. Guidelines for the Use of Wound Antiseptics

There are no global guidelines for the use of wound antiseptics. Unfortunately, apart from those presented below, there are no other guidelines in such databases as PubMed, Medline, Scopus, Web of Science, as well as the Google search engine. Therefore, consensus and guidelines, which appeared in recent years in Germany and Poland are of special importance: “Consensus on Wound Antisepsis: Update 2018 [28], and “Guidelines for local management of uninfected wounds, wounds at risk of infection and infected wounds—an overview of the available antimicrobial substances used in the treatment of wounds. Recommendations of the Polish Wound Treatment Society” [30]. A summary of the above can be found in the publication “Review of antimicrobial substances used in wound treatment based on the German consensus and Polish guidelines” [29]. It seems that the combination of this consensus and guidelines, based on the most recent research and data, could be a beneficial guide for the use of wound antiseptics for the whole of Europe.

**Figure 1:**
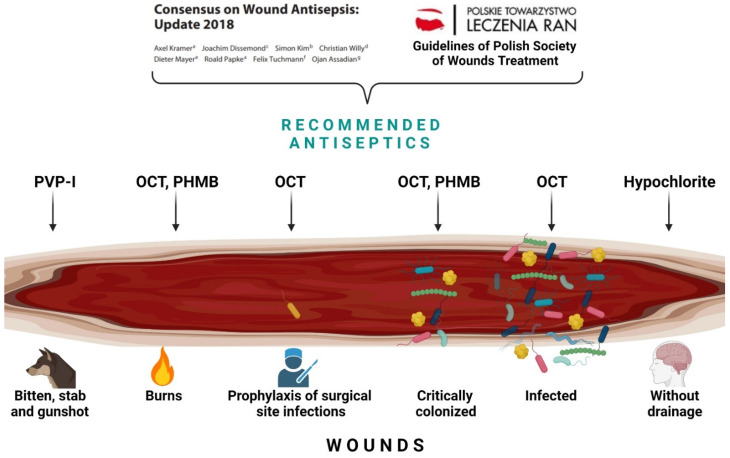
Antiseptics recommended according to the German Consensus and Polish Guidelines for the treatment of specific wounds [28,29,30].

Recent indications for the use of antiseptics in specific wounds, in accordance with the aforementioned guidelines and consensus, are presented in Figure 1 and Table 2.

## 6. Conclusions

There are many wound antiseptics available on the market. In the treatment of wounds, old antiseptics such as boric acid, ethacridine lactate, potassium permanganate, or hydrogen peroxide should be avoided. According to the newest guidelines, only the following antiseptics should be taken into account for wound treatment: OCT, PHMB, PVP-I, NaOCl and Nano-silver. It is essential to read the most up-to-date guidelines and product information for the antiseptics containing the above-mentioned active substances in order to maintain safety and preserve their efficacy.

## Figures and Tables

**Table 1:** Minimal inhibitory concentration (MIC) values for selected antiseptics against *Staphylococcus aureus* and *Pseudomonas aeruginosa* species.

Antiseptic	*S. aureus*	References	*P. aeruginosa*	References
	MIC (µg/mL)		MIC (µg/mL)	
OCT	0.25–4	[26,31,100,101]	1–8	[25,26,100,101]
PVP-I	7.8–512	[26,102,103,104]	2048–50,000	[105,106,107,108]
PHMB	0.25–8	[26,101,109,110,111,112]	2–32	[26,101,113,114]
NaClO	250–4063	[115,116,117,118]	1000–8192	[119,120,121]
Nano-silver	5–1350	[122,123,124]	0.3–27	[125,126]

**Table 2:** Summary of first and second choice measures for the treatment of specific wounds [28,29,30].

Indication	Anti-Bacterial Substance	
	First Choice	Second Choice
Wounds without drainage	Hypochlorite	-
Critically colonized wounds, wounds at risk of infection	PHMB (0.02%, 0.04%, 0.1%), OCT 0.05%	OCT/hypochlorite, silver
Bitten, stab and gunshot wounds	PVP-I	Hypochlorite
Wounds colonized or infected by MDRO	OCT	OCT 0.05%, PHMB, silver
Decontamination of acute and chronic wounds	PHMB, hypochlorite	-
Burns	PHMB, OCT 0.05%	OCT/hypochlorite
Rinsing the peritoneal cavity	Hypochlorite	-
Risk of PNS exposure	Hypochlorite	PVP-I
SSI prophylaxis	OCT	-

## Data Availability

Not applicable.
